# Prevalence and Impact of HIV Infections in Patients with Rheumatic Heart Disease: A Systematic Review and Meta-Analysis

**DOI:** 10.5334/gh.1265

**Published:** 2023-09-15

**Authors:** Evelyn N. Lumngwena, Dipolelo Mokaila, Olukayode Aremu, Patrick DMC Katoto, Jonathan Blackburn, Peter Zilla, Charles Shey Wiysonge, Ntobeko Ntusi

**Affiliations:** 1 School of Clinical Medicine, Faculty of Health Sciences, University of Witwatersrand, Johannesburg, ZA; 2 Centre for the Study of Emerging and Re-emerging Infections (CREMER), Institute for Medical Research and Medicinal Plant Studies (IMPM), Ministry of Scientific Research and Innovation, CM; 3 Cape Heart Institutes, Department of Medicine, Faculty of Health Sciences, University of Cape Town, ZA; 4 Division of Cardiology, Department of Medicine, Faculty of Health Sciences, University of Cape Town, ZA; 5 Cochrane South Africa, South African Medical Research Council, Francie van Zijl Drive, Parow Valley, 7501, Cape Town, ZA; 6 Centre for Tropical Diseases and Global Health, Catholic University of Bukavu, Democratic Republic of Congo; 7 Institute of Infectious Disease and Molecular Medicine, Faculty of Health Sciences, University of Cape Town, ZA; 8 Department of Integrative Biomedical Sciences, University of Cape Town, ZA; 9 Christiaan Barnard Division of Cardiothoracic Surgery, University of Cape Town, ZA; 10 World Health Organization Regional Office for Africa, Citédu Djoué, Brazzaville, CG; 11 Cape Universities Body Imaging Centre, Faculty of Health Sciences, University of Cape Town, Cape Town, ZA

**Keywords:** rheumatic heart disease, acquired HIV infections, vertical HIV transmission, prevalence

## Abstract

Socioeconomic factors such as poor health and poor nutrition in low- and middle-income countries (LMICs) may favour inflammatory reactions, thus contributing to the recurrence of rheumatic fever (RF) and thereby modifying trends in rheumatic heart disease (RHD). Apart from epidemiological studies, studies of HIV infections in RHD patients are limited. This systematic review synthesises data on the prevalence and impact of HIV infections or AIDS on RHD from PubMed, Scopus, Web of Science databases up to April 2021. The outcomes were managed using PRISMA guidelines.

Of a total of 15 studies found, 10 were eligible for meta-analyses. Meta-analysis found that 17% (95 % CI 8–33, I^2^ = 91%) of adults in cardiovascular disease (CVD) cohorts in Southern Africa are HIV positive. The proportion of RHD diagnosed among people living with HIV was 4% (95% CI 2–8, I^2^ = 79%) for adults but lower [2% (95% CI 1–4, I^2^ = 87%)] among perinatally infected children. Despite limited reporting, HIV-infected patients with RHD are prone to other infections that may enhance cardiac complications due to poor immunological control.

**PROSPERO registration number:** CRD42021237046.

## Introduction

Rheumatic heart disease (RHD) remains the predominant aetiology of acquired valvular heart disease in low- and middle-income countries (LMICs) [1], contrarily to degenerative and myxomatous diseases that are predominant in developed nations [2,3]. The clinical presentations of RHD vary in different geographical regions [4,5], probably due to differences in contributing socioeconomic factors and comorbidities [6,7]. Limited access to non-invasive diagnostic resources [8,9], inconsistent clinical spectrum and pathogenic mechanisms, and late clinical presentation of rheumatic fever (RF) symptoms [10,11,12,13] may further adversely affect disease history and prognostication. The development and progression rates to RHD differ between indigenous Australians and Africans, probably due to repeated episodes of RF or associated living conditions [14,15,16,17,18]. Furthermore, inflammatory reactions from other untreated infections may drive the recurrence of RF or RHD progression, thwarting the RHD clinical pathway [6,17,19].

Although surgical intervention remains the evidence-based corrective therapy for RHD, it is still not accessible to many, and, most importantly, the outcomes are not always favourable for advanced cases [20]. Multiple cardiac complications in late stages of RHD, such as pulmonary hypertension, atrial fibrillation, systolic dysfunction, severe biventricular dysfunction, stroke, endocarditis, and/or heart failure, may further complicate late-stage management of RHD [20,21,22]. This calls for the need for early diagnosis and management.

Apart from the traditional risks and known etiological causes of acquired heart diseases, other infections are increasingly shown to contribute to acquired cardiovascular (CV) complications [15,23,24]. Non-ischaemic cardiovascular complications are often seen at both early and late stages of HIV infection [21,22]. Late-stage complications of AIDS and toxic effects of HIV therapy (Figure 2) [18,25,26,27,28,29,30,31] may further aggravate complications of acquired heart diseases such as RHD [29,30]. The overlap of complications, such as pericarditis, hypertensive heart disease, pulmonary hypertension, and right-sided congestive cardiac failure in RHD and HIV-infected cohorts, may increase mortality when HIV-associated CV complications cumulate with RHD complications and modify progression [29,32].

Despite the overlapping burdens of HIV infections and RHD in sub-Saharan Africa (SSA) and other LMICs [18,23,24,29,30,31,33,34,35], little attention has been paid to the impact of HIV infection on RHD progression. Increased autoantibodies in HIV-infected RHD patients [36], or secreted antigenic proteins of HIV infection such as Tat proteins and gp120, may drive inflammation and B cell activation [37] that may also trigger RF recurrence and thereby alter RHD disease progression. Bacterial and non-bacterial endocarditis are also common in very severe RHD, potentially further complicating the management of RHD [38]. Similarly, the ever-increasing spectrum of emerging and re-emerging infections associated with HIV, or its products, may modify the course of RHD, too [39]. Additionally, metabolic complications associated with HIV infection, or the effect of HIV medications may also compound CV complications in RHD [14].

Beyond epidemiologic evidence, understanding whether HIV- associated inflammatory reactions modify pathogenetic mechanisms in the pathogenesis of RHD is important. Understanding the prevalence and impact of HIV co-infection with RHD may provide important supporting guidelines for monitoring, early intervention, or surgical management of severe cases with both conditions. We systematically synthesised the prevalence and impact of HIV infection in RHD cohorts. We anticipate that the outcomes of this review will inform further studies and guide recommendations for management of HIV-infected RHD patients in regions with heavy disease burden.

**Rationale:** The first episodes of RF take place early in life (ages 5–14 years), but RHD symptoms most often present years later in adolescents. Following RHD presentation, secondary prophylaxis may prevent progression to heart failure by reducing further autoimmune responses. Acute heart failure from multiple causes is frequent in the younger African population than in industrialised nations [15]. It is not known if autoimmune reactions due to HIV infection or its secreted products may further reactivate chronic inflammatory processes, potentially enhancing CV complications and the progression of RHD to heart failure.

**Impact:** Currently, there is a paucity of information on acquired HIV infection in patients with RHD or RHD in vertically acquired HIV-infected children. Lack of such information poses a barrier to establishing management guidelines. This study focuses on the status of HIV infection in RHD and highlights common CV complications shared by RHD and HIV.

### Methodology

**Figure 1:**
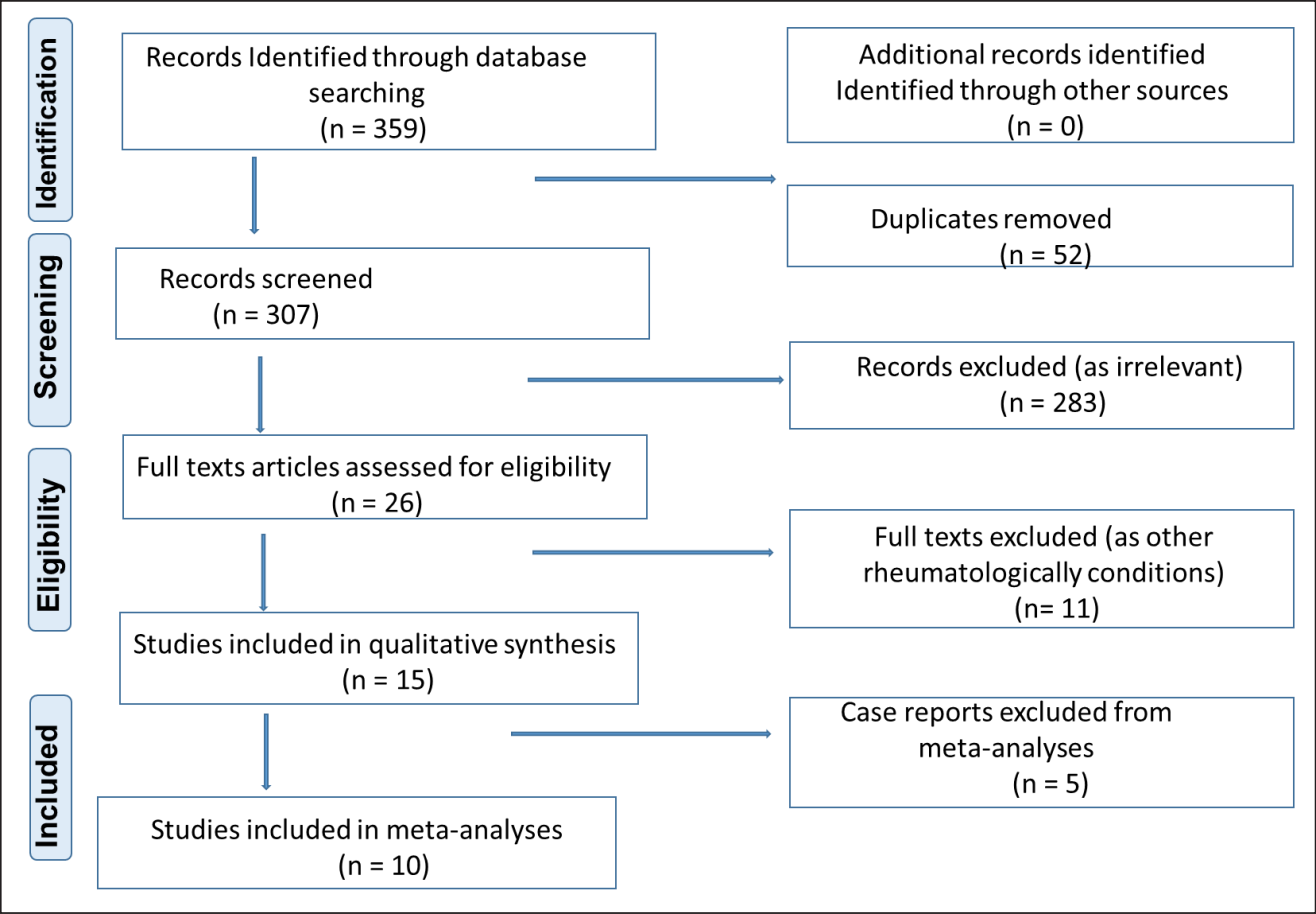
PRISMA flow diagram of study selection process for this review.

A study protocol was developed and registered on PROSPERO (PROSPERO #CRD42021237046), the database for monitoring of systematic reviews of clinical relevance. We conducted a systematic search of studies of RHD patients who were later infected with HIV or vice versa. The study was carried out and reported in keeping with the Preferred Reporting Items for Systematic Reviews and Meta-Analyses (PRISMA 2020) guidelines by Moher *et al* (2009) [40] (Figure 1) and Joanna Briggs Institute’s (JBI) recommendations for reporting of observational & epidemiological studies [41].

### Search Strategy

An advanced search was carried out in PubMed to build the search strategy. A search builder included medical subject headings (MESH) terms: #1 = **“Rheumatic heart disease” Rheumatic heart disease [All fields]** OR **“Rheumatic valvular heart disease”** OR **“Rheumatic mitral valve disease”** OR **“Rheumatic fever”** AND #2 = **“HIV infections”** OR **“HIV infection”** OR **“AIDS”** OR **“HIV”** were piloted. A combined search of “#3 = 1 AND #2” was piloted by ENL in PubMed. When #2 “HIV infections” or “AIDS” were piloted, no additional eligible article was found except multiple articles on HIV in other rheumatological diseases of the systemic autoimmune types (systemic lupus or rheumatoid arthritis) in HIV, which did not meet the criteria for RHD. The terms were then searched across the PubMed, Web of Science, and Scopus databases from 1981 to April 2021.

#### Selection criteria for eligible studies

We included all eligible observational and cross-sectional studies of RHD and HIV diagnosed in the same patient, irrespective of age and what part of the world they were conducted in, as recommend by the JBI CoCoPop methodology. In this process, basic and clinical studies, including case reports, case-controlled studies, cross-sectional studies, cohort studies, reviews, and systematic reviews, conducted in any setting and epidemiological reports on RHD subjects with confirmed diagnosis of HIV and RHD patients were considered. A study was eligible for inclusion if it was (a) primary research, (b) published in a peer-reviewed journal, (c) in English or French, and (d) a study for which RHD diagnosis was ascertained by imaging or any confirmatory method for diagnosis of RHD or by observation of commissural fusion or thickened mitral cusps or confirmed post-surgically and (e) a confirmatory test for HIV infection, being managed for HIV or not. Studies carried out on humans as well as post-mortem studies were included. Review papers and systematic reviews were only used to hand pick eligible studies from their reference list but were excluded from further analysis. Commentaries, editorials, book chapters and encyclopaedia entries, and studies of other cardiac valvular diseases in HIV patients, whereby the aetiology is not group A streptococcal GAS infection, were also excluded.

#### Data extraction and management

Two researchers (DM and AO) searched the databases independently and screened the search output (titles and abstracts) for eligible studies in English and French while ENL arbitrated. Full texts of eligible abstracts were further screened to ascertain eligibility when the two authors were in doubt. ENL accessed all the full-text publications of eligible studies using our predefined inclusion criteria to confirm eligibility. Full texts of potentially eligible studies written in French were to be translated by ENL before concluding for eligibility. All the studies found were saved in EndNote citation manager, which was also used to remove duplicates. An Excel sheet was designed for data extracted by ENL and a coding framework was developed by ENL and DM and a round of testing. Each of the two primary data-compiling authors independently extracted the titles, types of study, aims, methods used, results, and limitations, which were further compared and reported as on the PRISMA flow chart (Figure 1).

#### Indicators of quality and risk of bias assessment

We used the JBI’s critical appraisal checklist for studies reporting prevalence data [41]. We checked for clinical indication of HIV infection in primary RHD baseline participants and whether an HIV test was requested and excluded from meta-analyses if not tested. We further checked whether the HIV^+^ rheumatic cases had more cardiovascular and clinical complications compared to the HIV^–^ RHD cases. For baseline HIV-infected patients, we assessed if imaging (echocardiography or MRI) was used to confirm RHD diagnosis, if RHD diagnosis was done by a competent cardiologist, and if the sequence analyses for patients and controls were carried out by the same investigator or cardiologist. We further used the Newcastle-Ottawa Quality Assessment Scale (NOS) for assessing quality of non-randomised studies in meta-analyses for any bias in cases and control definition, selection, representation, and comparability (Sup Methods).

#### Data syntheses

A mixed synthetic approach was carried out. Qualitative data were synthesised in text and quantitative data (Table 1) meta-analysed. Thus, a two-staged analytic procedure where, firstly, the descriptive information, such as authors, study design, year of publication, and aim of the study, was extracted. Both quantitative and qualitative synthetic approaches were conducted for diagnosis and complications of HIV infections in RHD cohorts or RHD in people living with HIV (PLHIV). The meta-analysis of the content and results of each eligible study was the second approach.

**Table 1:** Characteristics of studies eligible for the systematic review.


STUDY	COUNTRY	AGE GROUP	PRIMARY DIAGNOSIS	NUMBER OF HIV AMONG THE RHD CASES	REFERENCE	COMMENTS

DiCarlo et al., 1989	United Sates	Adult	Case of IE	1 case (case study)	[48]	Excluded from meta-analysis

Radcliffe et al., 1991	United Kingdom	Adult, 25 years old	Case of IE	1 case (case study)	[49]	Excluded from meta-analysis

Mesquita et al., 1996	Brazil	Adult, 36 years old	A Case of RHD + HIV	1 case (case study)	[45]	Excluded from meta-analysis

Vohra et al., 2014	India	Adult, 21 years old	A case report of ARF + HIV	1 case (case study)	[47]	Excluded from meta-analysis

Sathekge et al., 2015	South Africa	28 years old	28-year-old HIV^+^ woman presented with productive cough, fever weight loss, and progressive dyspnoea class II to III for six months	1 case (case study)	[46]	Excluded from meta-analysis

1. Meel et al., 2017	South Africa	Adult, 30–60 years old	RHD cohort in Soweto	26 HIV/94 RHD	[34]	Included in meta-analysis

2. Schwartz, 2012	Botswana	Adults, 15–97 (mean 45 for RHDs)	Cardiomyopathy cohort	7 HIV/15 RHD (of a cohort of 179 CVD referrals)	[29]	Included in meta-analysis

3. Huck et al., 2016	Uganda	10–60 years old	Mixed: RHD+ and HIV^+^	21 HIV/115 RH adults	[36]	Included in meta-analysis

4. Sliwa et al., 2010	South Africa	Adults > 14 years old (97% > 20 years old)	RHD cohort	23 HIV^+^/344 RHD cases of various ages, 14–70 years, but only 3% of cases were 14–19 years, so mainly adults	[14]	Included in meta-analysis

5. Koegelenberg, 2003	South Africa	Adults (mean age 37.7)	IE study cohort	1 HIV^+^/36 RHD in a 47 IE cohort studied	[38]	Included in meta-analysis

6. Dobe et al., 2020	Mozambique	Adults, 18–75 years old	Study of HIV cohort	6 RHD/468 HIV cases (6 of 88 who had evidence of CVD in the 468 HIV cohort)	[50]	Included in meta-analysis

7. Sliwa et al., 2012	South Africa	Adult CV disease (mean age 41 years old)	Heart disease in HIV-infected adults	32 RHD/518 HIV adults	[51]	Included in meta-analysis

8. Hovis et al., 2016	Uganda	5–15 years old	HIV vertically acquired cohort	15 RHD/993 HIV children	[52]	Included in meta-analysis

9. Glearson et al., 2017	Uganda	7–13 years old	HIV vertically infected children	4 RHD/488 HIV children	[53]	Included in meta-analysis

10. Manafe et al., 2019	Mozambique	Children (median age 9 years old)	Vertically acquired HIV-infected children	1 RHD/47 HIV children	[54]	Included in meta-analysis


Eligible published reports selected for the systematic review and meta-analysis.

We found 15 eligible studies where HIV diagnosis was reported in the RHD patients (HIV^+^ RHD) or vice versa (RHD+ HIV). The number of cases and events, clinical morphological and functional characteristics of RHD phenotypes, and clinical parameters such as CD4+ counts and viral loads of HIV^+^ RHD HIV infection, where available, were extracted and compared to HIV^–^ RHD patients. Additionally, cardiac manifestations common to HIV populations, such as structural or functional cardiac muscle impairment, pulmonary hypertension, and pericardial effusion, when available, were extracted.

An R statistical software, *Meta Tools* (version 3.6.0; the R Foundation for Statistical Computing, Vienna, Austria) was used to perform the meta-analysis. *Metaprop* functions were used to generate prevalence data using the reference approach by Barendregt and colleagues [42]. Unadjusted prevalence was computed using the crude numerators and denominators from each study. The prevalence of HIV infection among RHD patients as well as that of RHD among all adults living with HIV infection and children with perinatally acquired HIV infection were estimated. Given that these are proportion studies with low proportion outcomes, the overall proportion was calculated using a logit transformation (by internally calling the *Metafor* function in the R package), and the 95 percent confidence interval (95% CI) was calculated using the Clopper-Pearson technique. We produced forest plots to illustrate the prevalence and 95% CI for each of the HIV and RHD studies, as well as the pooled prevalence obtained by integrating all studies. A subgroup analyses to determine the percentage of HIV infection or of RHD among patients living with HIV infection by country, age (adults vs children/adolescents), and CD4 count level was also conducted.

Before pooling the data, the variance of the study-specific prevalence was stabilised using the Freeman-Tukey double arcsine transformation to reduce the influence of studies with very small or extremely high prevalence estimates on the overall estimate. Although we presented pooled prevalence for both fixed and random effects models, since there was heterogeneity in each outcome, we interpreted our results using estimates from the random effects model (based on the DerSimonian and Laird technique) [43]. The chi-square test (based on Cochrane’s Q statistic) was used to assess heterogeneity, which was measured by H and I^2^ values. The I^2^ statistic calculates the proportion of total variance across studies that may be attributed to real between-study variations rather than chance (I^2^ values above 60% suggest the existence of significant heterogeneity) [43,44].

## Results

The systematic search output found two types of studies: CVD cohorts in which HIV infections were diagnosed in RHD patients, henceforth called HIV^+^ RHD patients, and HIV-infected cohorts in which RHD was diagnosed, henceforth referred to as RHD+ HIV patients. The search outputs and screening strategies are summarised in the PRISMA flow diagram (Figure 1). Five case reports of HIV-infected adults with rheumatic valvulitis were initially presented with infective endocarditis (IE) in injection drug users [45,46,47,48,49]). Another HIV^+^ RHD was diagnosed in an IE cohort of 47 patients in the Western Cape of South Africa [38].

Very few studies of echocardiograpically confirmed RHD cohorts of patients aged above 15 years that met the inclusion requested an HIV diagnosis. Further, cohorts of acquired HIV-infected adults and perinatally infected children were found with confirmed RHD, as summarised in Table 1. A meta-analysis was conducted in the two ways mentioned of all eligible studies except the clinical case reports.

### Description of included studies

Of the 15 eligible studies, 5 were cases studies. Of the 10 other studies included in meta-analyses, 3 were from adult cohorts with baseline RHD in a cardiovascular disease clinic that diagnosed HIV infection and 1 compared double RHD HIV^+^ cohort to those with one condition (RHD only or HIV only). Additionally, 3 studies were baseline adult HIV-infected cohorts which reported the proportion of RHD diagnoses using echocardiography. The other 3 studies were of children and early adolescents with perinatal HIV infection who were diagnosed with RHD during their management (Table 1).

### Pathophysiological impact

One study that compared phenotypic features of RHD found that a greater proportion of HIV^+^ RHD patients had severe mitral regurgitation MR compared to HIV^–^ RHD patients (50% vs. 23%, *p* = 0.015) [34]. More HIV^+^ RHD patients had type IIIa (restrictive) leaflet dysfunction, of whom 15% had mixed lesions (*p* = 0.05) compared to HIV^–^ RHD patients. Concomitant organic morphological tricuspid valve (TV) disease was more common in HIV^+^ RHD than in HIV^–^ RHD patients (50% vs 21%, *p* = 0.02). Right ventricular dilation was also slightly increased and more functionally impaired in HIV^+^ RHD compared to HIV^–^ RHD patients but did not reach statistical significance. Only 19% of HIV^+^ cases in the RHD cohort studied by Meel et al. were on antiretroviral therapy (ART) [34]. This is unlike the other studies of baseline HIV-infected adult cohorts, where most of the patients were ART treated [50,51], and the perinatally acquired HIV cohort, who commenced ART from birth [52,53]. Apart from the differences in echocardiographic features listed above, clinical presentations of HIV^+^ vs HIV^–^ RHD patients, such as CD4^+^ or viral loads that may characterise disease progression, were under-reported, with only one study finding significant higher viral loads and a trend towards lower CD4 counts, but only a few patients were evaluated [51]. None of the studies reported outcomes of any post-surgical follow-up, which is recommended for further research to understand the impact of HIV on left ventricle (LV) recovery. IE was diagnosed in all the case studies.

While Meel et al. study did not specifically compare CD4 counts for this HIV^+^/HIV^–^ RHD, the average proportion of infected HIV^+^ RHD patients remained 17% in each group and did not change, even when the pooled effect of CD4+ counts less than or greater than 300 cells/mm^3^ were compared, despite the heterogeneity and limited number of the studies found. A previous study found no association of LV and valvular structural changes on ECHO in HIV-infected adult cohorts with CD4+ counts below or above 200 cells/mm^3^ in the absence of RHD [55]. Thus, it is not known whether this measure of clinical status may affect the valve lesions or symptoms associated with RHD with advancing disease stage. However, most of the adult RHD/CV studies found in this synthesis did not report CD4 counts.

The HIV-infected children with RHD tended to be older (12 years vs 10) than those without RHD [52]. A lower CD4+ count was also found in the HIV^+^ RHD cohorts [52,53], but the small sample size (four RHD cases) did not give enough power for statistical significance. Apart from Huck’s study, where only 19% of HIV^+^ were on ART, there was no difference in ART medications, as most of the patients in the HIV^+^ cohorts were on ART. ART treatment was not different between the adult and the perinatally acquired HIV-infected cohorts (ART is administered from birth for positive children). In the perinatally infected cohorts, lower CD4+ counts and a lower percentage of children had viral loads control in the RHD-positive group compared to RHD-negative group [52], although the relation was not statistically significant due to the small sample sizes. This same trend was also seen in the RHD cohort in Soweto, although only few cases of viral load were measured [51].

An increase in anti-malondialdehyde (MDA) IgG and a decrease in IgM auto-antibody to the oxidation-associated phosphorylcholine (PC) epitopes was also reported in HIV-infected RHD compared to HIV^–^ RHD patients [36]. However, the extent to which these auto-antibody changes influence valvular lesions, LV dysfunction, and heart failure were not evaluated in this study.

### RHD cohort: proportion of HIV infections in RHD cohorts

**Figure 2:**
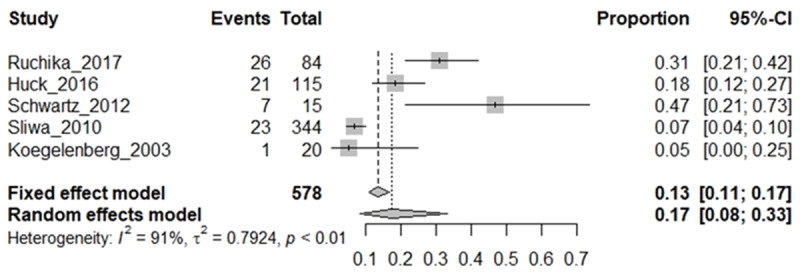
RHD cohort: proportion of HIV infections in RHD cohorts.

**Figure 3:**
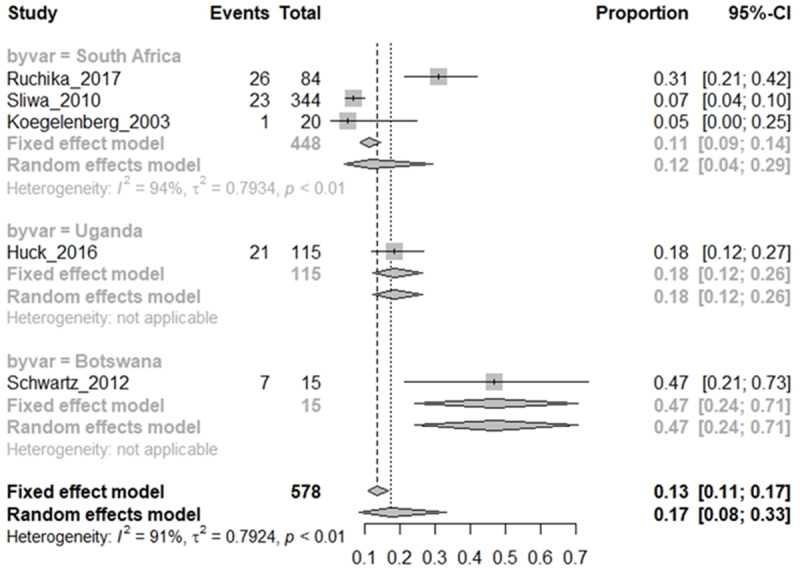
RHD Cohort: proportion of HIV infections in RHD cohorts by country.

A meta-analysis of the 10 eligible studies was conducted in R to determine the proportion of HIV^+^ RHD from the pooled eligible cohorts (Figure 2) and the proportion of RHD in the HIV-infected patient cohorts (Figure 3) of the total number of patients with each baseline condition and the confidence interval computed using the fixed effect model and random effect models. Moreover, few studies reported clinical measure of HIV disease state (CD4 and viral load) to evaluate the impact of RHD co-infection on clinical HIV infection.

Meta-analysis from four eligible CV/RHD baseline cohorts found that 17% of RHD patients in the CVD cohorts were diagnosed with HIV (HIV^+^ RHD), with a very wide 95% CI (8%–33%). There was, however, a substantial heterogeneity (I^2^ = 91%) in the study models due to the small sample size, evidenced by the wide 95% CI when the random effect model was considered (Figure 2). Given the heterogeneity of the studies, the random effect model was preferably interpreted, as it allows for inference of trends in proportions despite high heterogeneity.

Although the prevalence of HIV infection and RHD are high in LMICs, eligible studies of HIV infection in RHD were only found in Southern Africa, with South Africa representing the greatest number of eligible studies reported (Figure 3). As RHD and HIV prevalence are high among adolescents in South Africa, this may be an approximate of the incidence of HIV in RHD cohorts in high HIV-prevalent countries, including South Africa. Early screening for HIV among patients with RHD/CVD is recommended to inform early management decisions. However, the HIV^+^ RHD studies were most often conducted in adults presenting at tertiary CV specialist clinics/hospitals, and HIV screening was requested based on symptoms. This may be higher than the epidemiological evidence given the biased sampling sites and sampling guided by symptoms. Early diagnosis is recommended for RHD cohorts to allow for clinical decisions.

### HIV infection cohort: proportion of RHD in HIV-infected cohorts

**Figure 4:**
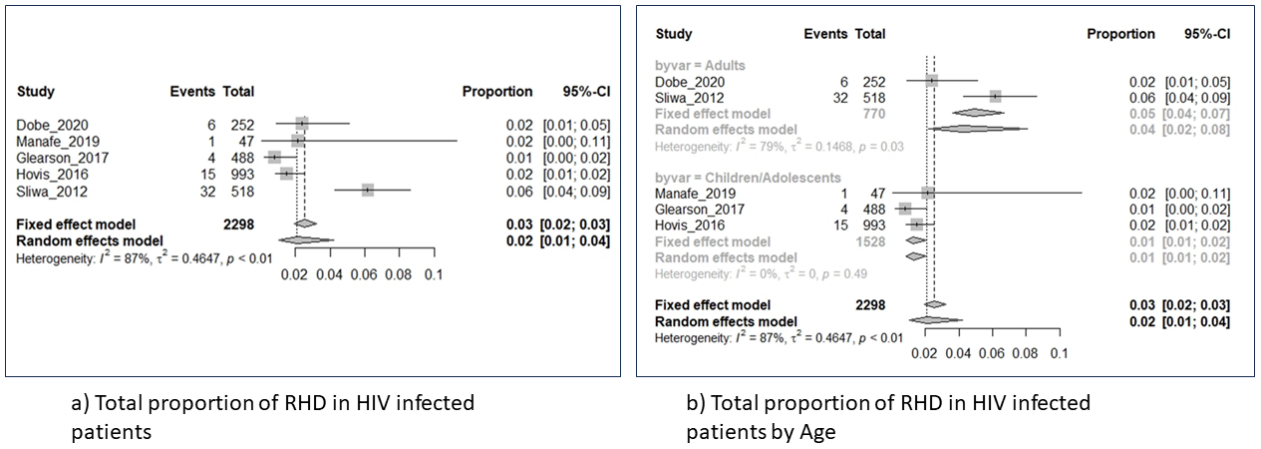
HIV infection cohort: proportion of RHD in HIV-infected cohorts, overall (4a) and by age (4b).

Pooling studies that used echocardiography to diagnose RHD among acquired HIV-infected adult cohorts and perinatally acquired HIV cohorts either before or after RHD infections showed differences in trend between adults and children (Figure 4a and 4b). An overall pooled proportion of 2% of both HIV-infected adults and perinatally infected children (range: 1%–4%; 95% CI) were diagnosed with RHD irrespective of age (Figure 4a). As shown in Figure 4b, a higher proportion of HIV^+^ adults (4%) were diagnosed with symptoms of RHD than perinatally HIV-infected children with RHD (2%). HIV-infected children diagnosed with RHD were older (12 years vs 10 years) than those with no RHD [52,53]. It is worth noting that perinatally HIV-infected children were on cotrimoxazole prophylaxis, a drug meant to prevent other infections. This may delay or prevent recurrence of RF episodes and thus prevent or delay RHD. However, as RHD develops over time post GAS infection and recurrent Acute RF episodes, age may effectively be a positive determinant of RHD development once confirmed GAS infection has taken place.

**Figure 5:**
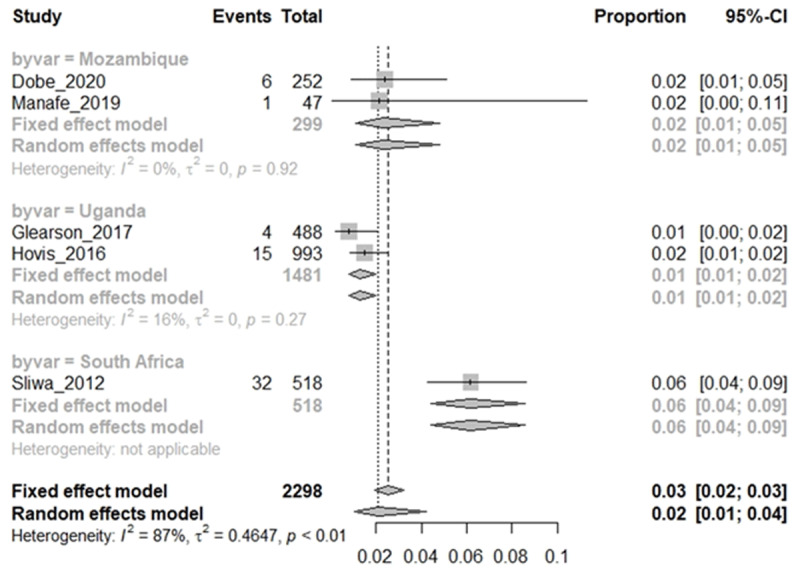
HIV infection cohort: proportion of RHD in HIV-infected cohorts by country.

Despite the heterogeneity, a high proportion of adult RHD patients in cardiac clinics (17%) were diagnosed with HIV infection in studies conducted in South Africa, Uganda, and Botswana (Figure 5). These countries all have high prevalence of both HIV and RHD in urban settings [14,29,34,36]. Reports of HIV prevalence among RHD patients in other SSA countries are lacking, which may provide a near epidemiological impact of HIV in CVD cohorts in SSA.

Only three counties, mainly in sub-Saharan Africa have reported co-infection of HIV and RHD (Figure 5), both of which have high prevalence of both HIV and RHD. Studies in other LMICs are either lacking or did not report RHD in HIV infections, while some studies of HIV CVD in other LMICs instead excluded RHD participants [56,57].

## Discussion

RHD is an acquired heart disease and a major cause of CV complications, leading to heart failure in youths and adolescents in LMICs [58]. CV complications associated with RHD include valvulitis, myocarditis, left ventricular hypertrophy and dysfunction, pericarditis, left atrial enlargement, atrial fibrillation, and pulmonary hypertension, leading to heart failure [58,59]. Following GAS infection and ARF, chronic inflammation and immune activation over many years drive repeated RF episodes, leading to the development of RHD. Poor environmental living conditions, other infections, and other pathogen products are drivers of sustained immune activation that may enhance autoimmune reactions, a known contributor to RHD development in genetically predisposed individuals following GAS infection [6,17]. During this period, these individuals are also exposed to risks of other infections, including HIV, especially given the high burden of both infections in many LMICs [60].

**Figure 6:**
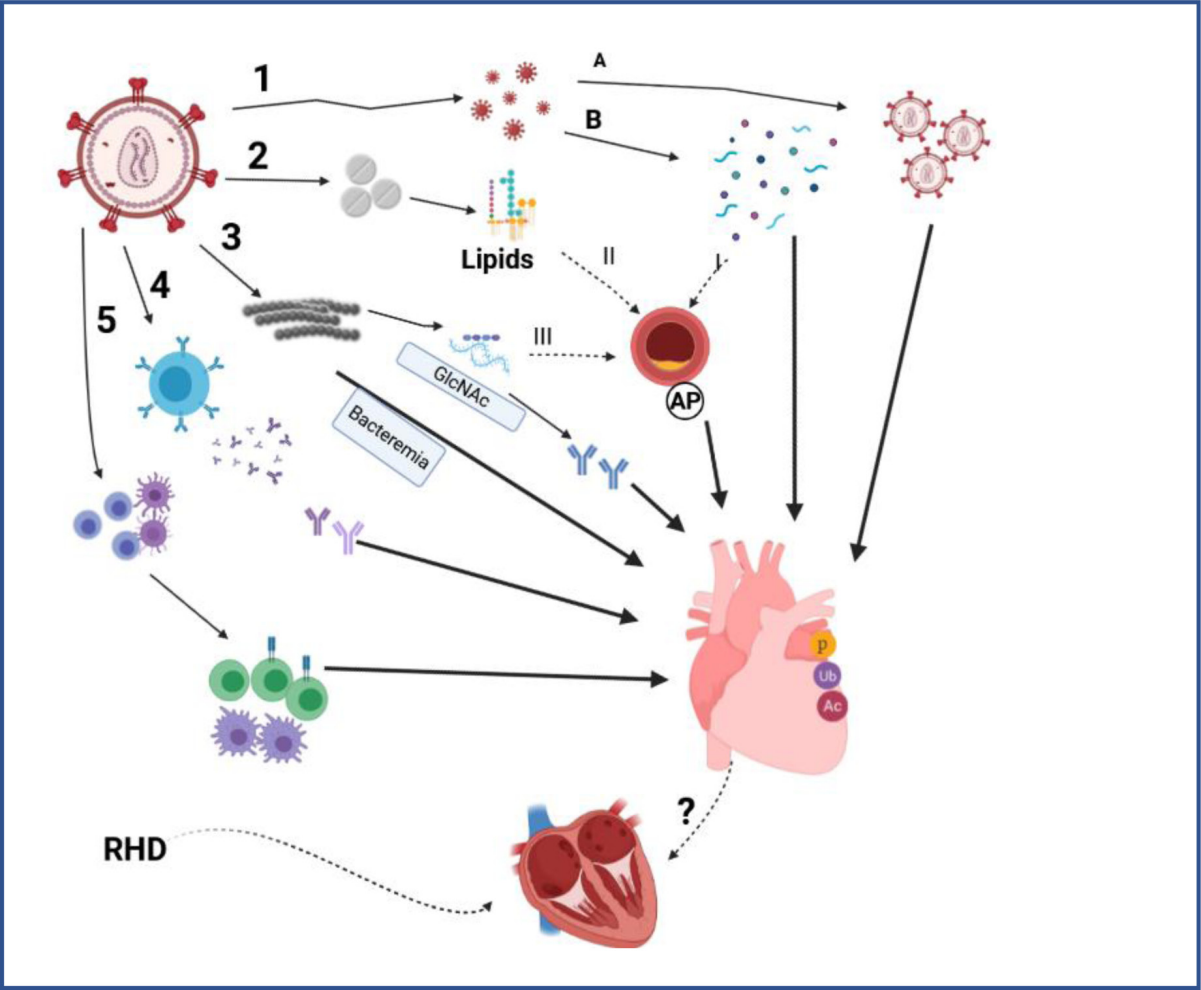
CV risk modification in HIV infection.

HIV infection is known to affect the CV systems through various mechanisms (Figure 6); viral replication and direct impact of the virus on the heart muscle may lead to myocarditis [61,62,63] (68) and generalised activation of the immune system and autoimmune disorders, even in elite HIV disease controllers [64]. Virus-induced systemic immune activation may release elevated levels of soluble mediators that may cause vascular endothelial dysfunction, leading to heart failure [65,66,67]. Viral products may cause atherosclerosis or chronic inflammation through activation of the adaptive immune mechanism, driving leukocyte infiltration into the myocardium and induction of myocarditis [30,65,67,68]. The effect of opportunistic pathogens such as *Toxoplasma gondii, Mycobacterium tuberculosis, Cryptococcus neoformans, Mycobacterium avium-intracellular* complex, *Aspergillus fumigatus, Candida albicans, Coccidioides, cytomegalovirus*, and *herpesviru*s types 1 and 2 and re-infection with GAS may also induce myocarditis or pericarditis or may secrete antigenic products and metabolites or release proteins whose epitopes may induce antibody responses that may cross-react with myocardium or endocardium and cause myocarditis or endocarditis [31,62,65,69,70,71,72]. These may induce valvulitis and hence further aggravate RHD. In fact, cardiac-induced autoimmune reactions with elevated anti-myosin antibodies similar to those found in RHD- were also reported in HIV-associated myocarditis (Figure 6) [63]. Furthermore, metabolic abnormalities (lipodystrophy and hyperlipidaemia) caused by HIV medications may also lead to atherosclerosis, modify CVD risks, and cause coronary artery occlusion, further aggravating RHD-induced CV complications [25,27,28,30,65,73].

Through these multiple mechanisms, HIV may further compound CV complications of RHD, such as endocarditis, myocarditis, LV dysfunction, pulmonary hypertension, and pericarditis [32,51]. These may lead to poor RHD management outcomes [74,75,76]. In fact, left ventricular dysfunction, a common complication from valvular malfunction in RHD, is also commonly associated with advanced HIV/AIDS, further complicating the management of RHD [28].

In chronic HIV infection, gut mucosal barrier dysfunction may also allow translocation of bacteria and fungi and enhance dysbiosis [77], which may stimulate the release of soluble mediators that affect the CVD system or induce cross-reactive Immune Responses to compound those of RHD [78,79].

Viral replication and direct impact of viruses on myocarditis (A). Viral products induce immune responses leading to myocarditis or atherosclerosis and CV disease (B). 2. Metabolic disorders (with lipodystrophy) exacerbated by the effect cART (HAART) may cause atherosclerosis and probably subsequent coronary artery disease or further modify other CVD risks. 3. HIV-induced physiological changes may favour survival of other pathogens, tipping the balance of even commensal bacteria; dysbiosis in HIV availing bacterial cell wall GlcNAc protein epitopes, inducing antibodies that may cross-react with the myocardium or endocardium and aggravate RHD-induced myocarditis. If anti-citrulline antibodies and other modifications to oral ligament proteins infected by oral/gingival bacteria cross-react with cardiac tissues, they may enhance autoimmune inflammatory responses affecting the heart. These mediate physiological changes and immune activation of chronic inflammation involving both humoral immune (4) and lymphocyte responses (5), driving leukocyte infiltration into the heart and inducing myocarditis. (Figure created in www.biorender.com).

As HIV-associated opportunistic infections like tuberculosis (TB) also further complicate HIV-associated pericarditis in RHD disease, further management recommendations are required, as most of these patients have been excluded from previous clinical trials [80]. Both HIV and RHD are highly prevalent in adolescents in SSA and other LMICs [58,81]. It will thus not be surprising if HIV infections reactivates the de novo presentation of symptoms of some acquired heart diseases, including RHD, allowing for quicker valve degeneration [51]. This may impact progression of the phenotypes of ARF patients that naturally took longer to progress to complete RHD [4]. Early management of HIV may also be required to achieve viral and immunological controls to allow for optimal post-surgery recovery [82].

An unacceptably high rate of RHD overlaps with perinatal HIV transmission in Mozambique [54], Uganda [52,53], and many LMICs. HIV-infected as well as exposed seronegative children may also have an entirely different response to GAS infection and RHD and may therefore require deeper investigations.

The present systematic review found a higher proportion of RHD among adults living with HIV than in vertically infected HIV^+^ infants [52,53]. The proportion of HIV-infected RHD infants was also lower compared to that of normal RHD among same aged children in similar settings [7] and lower than the HIV^+^ adult prevalence in Southern Africa [51]. This was in support of the Gleason et al. (2017) study that found a low prevalence in Ugandan HIV-infected children. Children with perinatally transmitted HIV, especially in this cohort, were administered ART and cotrimoxazole prophylaxis for opportunistic infections [52]. Children distant from a hospital had higher RHD prevalence in other areas of Uganda, suggesting a role for close medical monitoring, which includes cotrimoxazole administration [6]. This confirms findings from another study that socioeconomic (SE) and environmental factors enhance the risks of RHD and that close management reduced RF occurrence and thus RHD [6,17]. Low SE factors probably increase stress levels that allow other endemic infections to thrive and may enhance autoimmune inflammation, such as the recurrence of RF episodes/RHD progression, especially when there is lack of treatment resources, explaining the increased prevalence in areas farther from the hospitals in Uganda [6,17].

Although the prevalence of RHD was reduced in HIV^+^ compared to HIV seronegative cohorts, HIV^+^ RHD cases tended to have less virologic controls and lower immune status and were much older than those with no RHD, although both were followed with ART and cotrimoxazole prophylaxes. RHD may take years to develop post ARF episode. A close follow-up study over years would be necessary to ensure maintenance of this low RHD prevalence among HIV^+^ children. This low prevalence also needs to be confirmed in similar settings in other LMICs.

A high prevalence of acquired HIV was found among Botswanans [29] and South Africans with an RHD adult cohort in a tertiary CVD centre [14,34,51]. Many other RHD cohorts have not reported the HIV positivity, nor was HIV diagnosed. For example, although a study in Durban, South Africa, reported 31/95 HIV cases in a cohort of 77/95 RHD pregnant women, these numbers clearly showed an overlap, but the exact number of RHD HIV+ was not reported. In fact, some patients with clinical indications (18/95) declined consent for HIV testing in this cohort [83]. Although these have only been reported in SSA, studies of HIV-associated CV disease in India reportedly excluded patients with RHD and other acquired heart disease in their cohort, as they aimed to find CV complications of HIV [56,57]. This further suggests recommending the reporting of HIV^+^ RHD+ in other LMICs apart from SSA, as the older HIV-infected age groups with acquired heart disease (RHD valvulitis) may require a multidisciplinary care team.

One study found HIV+ RHD patients to present with more severe MR compared to HIV^–^ RHD patients [34] and type IIIa (restrictive) leaflet dysfunction, and some patients had mixed lesions. Concomitant organic morphological tricuspid valve disease was more common in HIV^+^ RHD than in HIV^–^ RHD patients. RV was slightly dilated and more impaired in HIV^+^ RHD compared to HIV^–^ RHD patients, but was not statistically significant. Only one-fifth of the HIV^+^ cases in the adult RHD study cohort were on ART [34]. This is unlike the other studies with baseline HIV, where most of the patients are ART treated.

Increased autoantibodies, indicative of oxidative stress conditions, that have been associated with free radical formation in various health conditions, including CVD and other autoimmune conditions, such as Alzheimer’s, asthma, and cancers [84,85], were probably an indication of increased stress in RHD^+^ HIV infections [36]. HIV or other infections may co-activate nearby genes, epigenetically modulating inflammatory reactions that may influence RHD complications. A recommendation will be that as a requirement for management guidelines, related studies report CV complications for HIV^+^ RHD patients.

Apart from differences in phenotypic echocardiographic features, as listed above, there was limited reporting of clinical presentations of HIV^+^ vs HIV^–^ RHD patients, such as CD4+, or viral loads to characterise disease progression as recommended. None of the studies also reported outcomes of post-surgical management of HIV^+^ RHD, which may be recommended to understand the impact of HIV on LV recovery. Most of the studies were not designed to compare clinical LV or RV functional impairment, pericarditis, left atrial enlargement, atrial fibrillation, or pulmonary hypertension between RHD HIV^+^ positive and RHD HIV^–^ negative cases, which will be recommended in future studies.

## Conclusion

There is a high burden of RHD in LMICs, where it is the major cause of heart failure in youths and adolescents in these settings [86,87]. A majority of HIV-infected cases also resides in these LMICs, a greater proportion of whom are equally young and adolescents [88]. HIV patients are also predisposed to a whole range of non-ischaemic, coronary or CV conditions that may aggravate the CV complications in RHD. Patients with acquired heart diseases of other aetiologies are also predisposed to HIV infection that may compound the non-ischaemic or acquired cardiovascular complications. However, few studies reported a non-significant difference in short-term surgical outcomes of HIV-associated CV complications [89]. It will be important to diagnose these early to inform management strategies. For RHD, recommended management may include both non-surgical as well as cardiac surgical procedures, ranging from valve surgery to even a heart transplant. Most importantly, it is useful to understand the impact of both to better inform management policies. A competent immune system and viral suppression are recommended during surgical management of RHD. Early diagnosis and virological control are recommended to prevent HIV complications and thus delay RHD progression to heart failure.

## Strengths and limitations

- Paucity of literature on HIV infection in RHD/acquired CVD patient cohorts or RHD in HIV-infected cohorts.
- The outcome of this study highlights existing gaps in reporting of HIV infections in RHD patients and the impact of acquired or vertical HIV infection on RHD progression.
- This study may motivate further research to improve benefits of cardiac surgery in CVD populations/RHD with secondary HIV infection and management of post-surgical complications in severely immunocompromised RHD patients.
- The serological and immune status of the patients for corrective surgical therapy for RHD is important for the cardiologist and patient management.
- The extent of CV complications like pulmonary hypertension (PH), myocarditis, and LV dysfunction common to both HIV and RHD are not reported in the studies.

## Data Accessibility Statements

All extracted data from eligible studies are included in the manuscript (and its supplementary files).

## Additional Files

The additional files for this article can be found as follows:

10.5334/gh.1265.s1 Sup Method. NOS manual.

10.5334/gh.1265.s2 Sup Table 1. Characteristics of primary RHD cohort.

10.5334/gh.1265.s3 Sup Table 2. Characteristics of primary HIV cohort.

10.5334/gh.1265.s4 Sup Table 3. PRISMA 2020 Checklist.

## Protocol Registration

The protocol for this systematic review was prepared according to PRISMA guidelines and registered in the database for management of systematic reviews of clinical impact, PROSPERO # CRD42021237046. Protocol can be assessed from the PROSPERO database.
