# Supplementary material for: Prevalence and Impact of HIV Infections in Patients with Rheumatic Heart Disease: A Systematic Review and Meta-Analysis
Source: Glob Heart. 2023 Sep 15;18(1):49. doi: 10.5334/gh.1265 (PMC10503531; doi:10.5334/gh.1265)
Supplement: Sup Table 1. — Characteristics of primary RHD cohort. [file gh-18-1-1265-s2.pdf]

Supplementary Files 1

ID	Author	Year	RHD.Cohort	Country	Age.average
1	Ruchika_2017	2017	Yes	South Africa	44
2	Huck_2016	2016	Yes	Uganda	40
3	Schwartz_2012	2012	Yes	Botswana	
4	Sliwa_2010	2010	Yes	South Africa	42
5	Koegelenberg_20	2003	Yes	South Africa	38

	Adults	Event	Sample.size	Proportion	CD4.Averag	CD4.below.300
	Yes	26	84	0.30952381		Unknow
	Yes	21	115	0.182608696	493	CD4>=300 cells/mm^3
	Yes	7	15	0.466666667	299	CD4<300 cells/mm^3
	Yes	23	344	0.066860465	298	CD4<300 cells/mm^3
	Yes	1	20	0.05	437	CD4>=300 cells/mm^3
