# Supplementary material for: Prevalence and Impact of HIV Infections in Patients with Rheumatic Heart Disease: A Systematic Review and Meta-Analysis
Source: Glob Heart. 2023 Sep 15;18(1):49. doi: 10.5334/gh.1265 (PMC10503531; doi:10.5334/gh.1265)
Supplement: Sup Table 2. — Characteristics of primary HIV cohort. [file gh-18-1-1265-s3.pdf]

Supplementary Files 2

ID	Author	Year	HIV.Cohort	Country	Age.average	Age
1	Dobe_2020	2020	Yes	Mozambique	39	Adults
2	Manafe_2019	2019	Yes	Mozambique	9	Children/Adol
3	Glearson_2017	2017	Yes	Uganda	8	Children/Adol
4	Hovis_2016	2016	Yes	Uganda	9	Children/Adol
5	Sliwa_2012	2012	Yes	South Africa	40	Adults

Children.Adolescent	Adults	Event	Sample.size	Proportion	CD4.Average
	Yes	6	252	0.023809524	516
Yes		1	47	0.021276596	900
Yes		4	488	0.008196721	801
Yes		15	993	0.01510574	1140
	Yes	32	518	0.061776062	298

CD4.below.500
CD4<500 cells/mm^3
CD4>=500 cells/mm^3
CD4>=500 cells/mm^3
CD4<500 cells/mm^3
CD4>=500 cells/mm^3
